# Identification of a novel trigger complex that facilitates ribosome-associated quality control in mammalian cells

**DOI:** 10.1038/s41598-020-60241-w

**Published:** 2020-02-25

**Authors:** Satoshi Hashimoto, Takato Sugiyama, Reina Yamazaki, Risa Nobuta, Toshifumi Inada

**Affiliations:** From the Graduate School of Pharmaceutical Sciences, Tohoku University, Sendai, 980-8578 Japan

**Keywords:** Ribosomal proteins, Ubiquitylation, Translation, Ribosome

## Abstract

Ribosome stalling triggers the ribosome-associated quality control (RQC) pathway, which targets collided ribosomes and leads to subunit dissociation, followed by proteasomal degradation of the nascent peptide. In yeast, RQC is triggered by Hel2-dependent ubiquitination of uS10, followed by subunit dissociation mediated by the RQC-trigger (RQT) complex. In mammals, ZNF598-dependent ubiquitination of collided ribosomes is required for RQC, and activating signal cointegrator 3 (ASCC3), a component of the ASCC complex, facilitates RQC. However, the roles of other components and associated factors of the ASCC complex remain unknown. Here, we demonstrate that the human RQC-trigger (hRQT) complex, an ortholog of the yeast RQT complex, plays crucial roles in RQC. The hRQT complex is composed of ASCC3, ASCC2, and TRIP4, which are orthologs of the RNA helicase Slh1(Rqt2), ubiquitin-binding protein Cue3(Rqt3), and zinc-finger type protein yKR023W(Rqt4), respectively. The ATPase activity of ASCC3 and the ubiquitin-binding activity of ASCC2 are crucial for triggering RQC. Given the proposed function of the RQT complex in yeast, we propose that the hRQT complex recognizes the ubiquitinated stalled ribosome and induces subunit dissociation to facilitate RQC.

## Introduction

Cells have evolved various quality control mechanisms to guarantee accurate gene expression^1–4^. Ribosome stalling induces quality control mechanisms for mRNA, referred to as No-go decay (NGD)^5–7^, as well as for protein, referred to as ribosome-associated quality control (RQC)^8–11^. RQC is conserved throughout species and consists primarily of four steps: (i) recognition of abnormal ribosome stalling; (ii) ubiquitination of specific residue(s) on the stalled ribosome; (iii) dissociation of ribosome into 40S and 60S subunits; and (iv) degradation of the nascent polypeptide on the 60S subunit^2,4^. In the first step of RQC, the stalling of a ribosome at a specific sequence results in the formation of a di-ribosome (disome), which consists of the leading stalled ribosome and the following collided ribosome^5,6,10^. Cryo-EM structural analysis has shown that the leading stalled ribosome is in the POST-state, with an empty A-site, whereas the colliding ribosome is in a rotated state with hybrid tRNAs^6,8,10^. In the second step, the RING-type E3 ubiquitin ligase Hel2 recognizes the ribosome collision and ubiquitinates ribosomal protein uS10 (in yeast, at residue(s) K6/8)^8^. In the third step, ubiquitinated ribosomes are dissociated into 40S subunits and 60S ribosome-nascent chain complexes (60S-RNCs), leading to subsequent RQC reactions. We recently proposed a model in which these ubiquitinated ribosomes are targeted by the RQT complex^8^. The RQT complex is composed of three proteins: RNA helicase Slh1(Rqt2), ubiquitin-binding protein Cue3(Rqt3), and zinc-finger domain-containing protein yKR023W(Rqt4). The ubiquitin-binding activity of Cue3 and the ATPase activity of Slh1 are crucial for triggering RQC^8^. After subunit dissociation, Rqc2 binds to tRNA at the subunit interface of 60S-RNCs to prevent re-association of the 40S subunit, and facilitates binding of Ltn1 to the 60S-RNCs^12^. Ltn1, a RING-type E3 ubiquitin ligase, ubiquitinates nascent peptides on the 60S subunit^13^. The ubiquitinated peptides are released by tRNA endonuclease Vms1^14^ and extracted by Cdc48 for degradation by the proteasome^15^.

**Figure 1:**
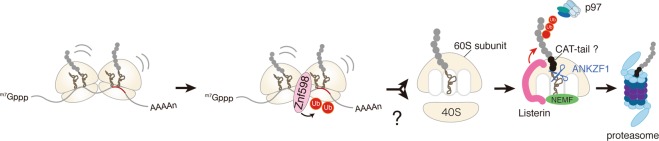
Proposed model for the Ribosome-associated quality control (RQC) pathway in mammals. A stalled ribosome collides with the following ribosome, and ZNF598 ubiquitinates the collided ribosomes. In yeast, it was proposed that the RQT complex dissociates the ubiquitinated ribosome(s) into subunits but the complex that recognizes and splits ubiquitinated ribosome(s) is unknown in mammals. Nascent peptide on the 60S subunit is ubiquitinated by Listerin, and released from the ribosome by ANKZF1 and p97. Then, the ubiquitinated polypeptides are degraded by the proteasome.

In mammals, ZNF598, an ortholog of Hel2, ubiquitinates eS10 of collided disome at K138/139^9,11,16^; its N-terminal RING domain is required for RQC. After ubiquitinated ribosomes are dissociated into subunits, NEMF, the mammalian ortholog of Rqc2, binds to tRNA at the interface of 60S-RNCs^17^. NEMF also facilitates binding of LTN1 to 60S-RNCs^17^. LTN1 polyubiquitinates nascent peptide with a K48-linkage, and TCF25, a mammalian homolog of yeast Rqc1, accelerates this preferential linkage formation by restricting the elongation of K63-linkages^18^. Ubiquitinated peptides are released from 60S-RNCs by the tRNA endonuclease ANKZF1^18,19^ and extracted by p97 for degradation by the proteasome^15^ (Fig. 1).

Despite the identification of various RQC factors in mammals, the factors that recognize ubiquitinated stalled ribosomes and promote their dissociation into subunits remain mostly unknown. We previously reported that ASCC3 is required for RQC induction^8^. A recent study showed that ASCC2 and ASCC3 bind to the ribosome and mitigate the toxic effects of stalling induced by PF8503^20^. The ATP-dependent helicase ASCC3 is an ortholog of Slh1(Rqt2)^8^, and the ubiquitin-binding protein ASCC2 has significant sequence homology to yeast Cue3(Rqt3), implying the existence of a human RQT (hRQT) complex. Here, we show that the hRQT complex is composed of ASCC3/2-TRIP4 and facilitates RQC in a manner dependent upon the ATPase activity of ASCC3 and the ubiquitin-binding activity of ASCC2. We found that ASCC1 is not an essential component of the hRQT complex, indicating that the hRQT complex is distinct from both the ASCC complex involved in DNA alkylation repairing and the ASC-1 complex that serves as a transcriptional coactivator. Based on these results, we propose that the newly identified hRQT complex facilitates RQC by dissociation of the ubiquitinated ribosomes into subunits in mammalian cells.

## Results

### Analysis of the domains of ZNF598 required for RQC

**Figure 2:**
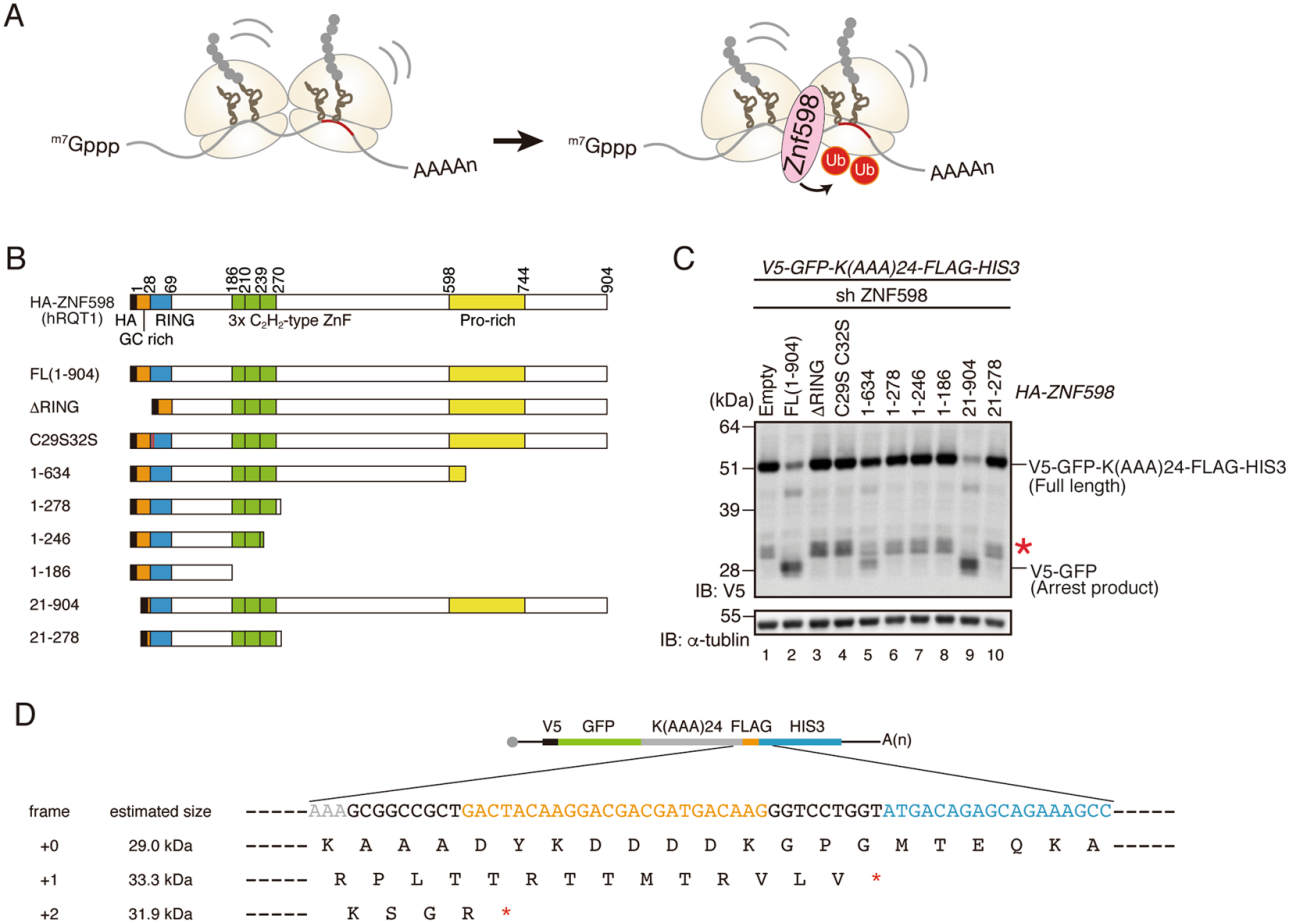
Domain mapping of ZNF598 in RQC. **(A)** ZNF598 recognizes the collided ribosomes and ubiquitinates ribosome proteins. **(B)** Schematic drawing of the series of ZNF598 mutants. **(C)** ZNF598 KD cells were co-transfected with *V5-GFP-K(AAA)*_*24*_*-FLAG-HIS3* reporter and the indicated *HA-ZNF598* mutants. Proteins were detected by western blotting with anti-V5 antibody, and the full-length (V5-GFP-K(AAA)_24_-FLAG-HIS3) and arrest products (V5-GFP) were detected. Asterisk indicates the frameshift product. Cropped blots were displayed. Full uncropped blots are available in Supplemental Fig. S2. The blots are representative of three independent experiments. **(D)** Estimated sequence and product size of frameshift products in *V5-GFP-K(AAA)*_24_*-FLAG-HIS3* reporter.

To determine which part > region of ZNF598 is required for the induction of RQC (Fig. 2A,B), we constructed a series of mutants, focusing on the characteristic domains of ZNF598. ZNF598 contains an N-terminal GC-rich region, followed by a RING domain, three C2H2-type zinc-finger domains, and a proline-rich motif at the C-terminus (Fig. 2B). We co-transfected these mutants along with the *V5-GFP-K(AAA)*24*-FLAG-HIS3* reporter into ZNF598 knockdown (KD) cells constitutively expressing shRNA against ZNF598, and then monitored RQC induction by western blotting with anti-V5 antibody. We evaluated RQC by assessing the levels of the full-length and arrest products: ZNF598 is functional, the level of the full-length product will decrease, and the levels of the arrest products will increase. Given that RQC excludes arrest products, the arrest products should not be observed when functional full-length ZNF598 is expressed. Because we tested the function of ZNF598 in cells overexpressing a poly(A)-coding reporter, we suspected that arrest products were produced in excess and could not be completely cleared by RQC (Fig. 2C, lane 2). We observed the frameshift products (Fig. 2C, asterisk at lanes 1, 3–8 and 10), which were in accordance with previous reports^11,16^, and the size of the frameshift products was also as expected (Fig. 2D). The RING domain deletion mutant (ΔRING) and its conserved cysteine residues mutant (C29S/C32S) did not induce RQC (Fig. 2C, lanes 3 and 4), whereas RQC was partially induced by the Pro-rich region trimmed mutant (1–634) but not the Pro-rich region deletion mutant (1–278) (Fig. 2C, lanes 5 and 6). Deletion mutants lacking the C2H2-type zinc-finger domain (1–246, 1–186) did not induce RQC (Fig. 2C, lanes 7 and 8). Moreover, the deletion of the N-terminal GC-rich region (21–904 and 21–278) had no effect on the induction of RQC (Fig. 2C, lanes 9 and 10). Finally, we confirmed that these phenotypes were not dependent on the expression levels of ZNF598 mutants (Fig. S1A). Based on these results, we conclude that the cysteine residues (C29, C32) within the RING domain and C-terminal regions containing the zinc-finger and Pro-rich region are both essential for induction of RQC.

### The human RQC-trigger (hRQT) complex consists of ASCC3, ASCC2, and TRIP4

**Figure 3:**
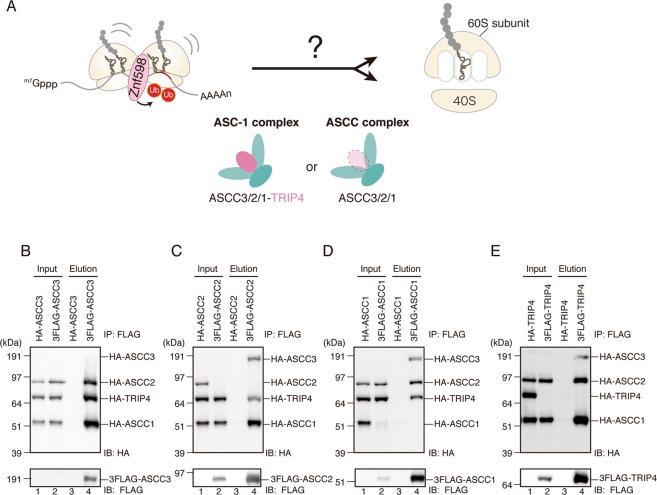
ASCC3/ASCC2/ASCC1/TRIP4 form a complex. **(A)** Schematics of the ASC-1 and ASCC complexes, and their components. These are possible candidates for the hRQT complex that recognizes and dissociates ubiquitinated ribosome(s). **(B–E)** FLAG or HA-tagged ASCC3, ASCC2, ASCC1, and TRIP4 were transfected into HEK293T cells, and the total lysates (input) were immunoprecipitated by anti-FLAG antibody beads. FLAG-tagged proteins were eluted by the FLAG peptides (elution). Both the input and elution fractions were analyzed by western blotting with antibodies against FLAG and HA. Cropped blots were displayed. Full uncropped blots are available in Supplemental Fig. S3. Blots shown are representative of three independent experiments.

We previously reported that an ortholog of yeast Slh1, ASCC3, is required for RQC^8^. In addition, a recent study suggested the involvement of ASCC3 and ASCC2 in co-translational quality control^20^. ASCC3 was originally identified as a component of the activating signal cointegrator 1 (ASC-1) complex, which consists of ASCC3, ASCC2, ASCC1, and TRIP4/ASC-1 (Fig. 3A)^21,22^. The ASC-1 complex promotes transactivation by serum response factor (SRF), activating protein 1 (AP-1), and nuclear factor κB (NF-κB) through direct binding to SRF, c-Jun, p50, and p65^22^. ASCC3 is also a component of the ASCC complex, which is composed of ASCC3, ASCC2, and ASCC1 (Fig. 3A)^22^. ASCC3 binds to the demethylation enzyme ALKBH3 and repairs alkylated DNA^23^. Proper recruitment of the ASCC repair complex requires recognition of K63-linked poly-ubiquitin chains by the CUE (coupling of ubiquitin conjugation to ER degradation) domain of ASCC2^24^. ASCC1 binds to ASCC3 and mediates the proper recruitment of the ASCC complex during alkylation damage^25^. TRIP4 (TR-interacting proteins) is a transcription coactivator in the nucleus and is also involved in trans-repression between nuclear receptors and AP-1 or NF-κB^22,26^. TRIP4 contains an autonomous transactivation domain harboring a zinc-finger motif, which also serves as a binding site for TATA-binding protein (TBP), TFIIA, SRC-1, CBP/p300, and nuclear receptors^21^.

To identify the components of the hRQT complex, we first examined the interaction between ASCC3, ASCC2, ASCC1, and TRIP4 in HEK293T cells. In these experiments, a FLAG-tagged bait protein (i.e., one of the aforementioned proteins) was overexpressed with HA-tagged prey (the rest of the proteins listed above) and immunoprecipitated with anti-FLAG antibody (Fig. 3B–E). In all cases, immunoprecipitation of FLAG-tagged proteins resulted in co-purification of the remaining HA-tagged proteins. These results indicate that ASCC3/2/1 and TRIP4 form a complex, consistent with previous reports^21,22^.

**Figure 4:**
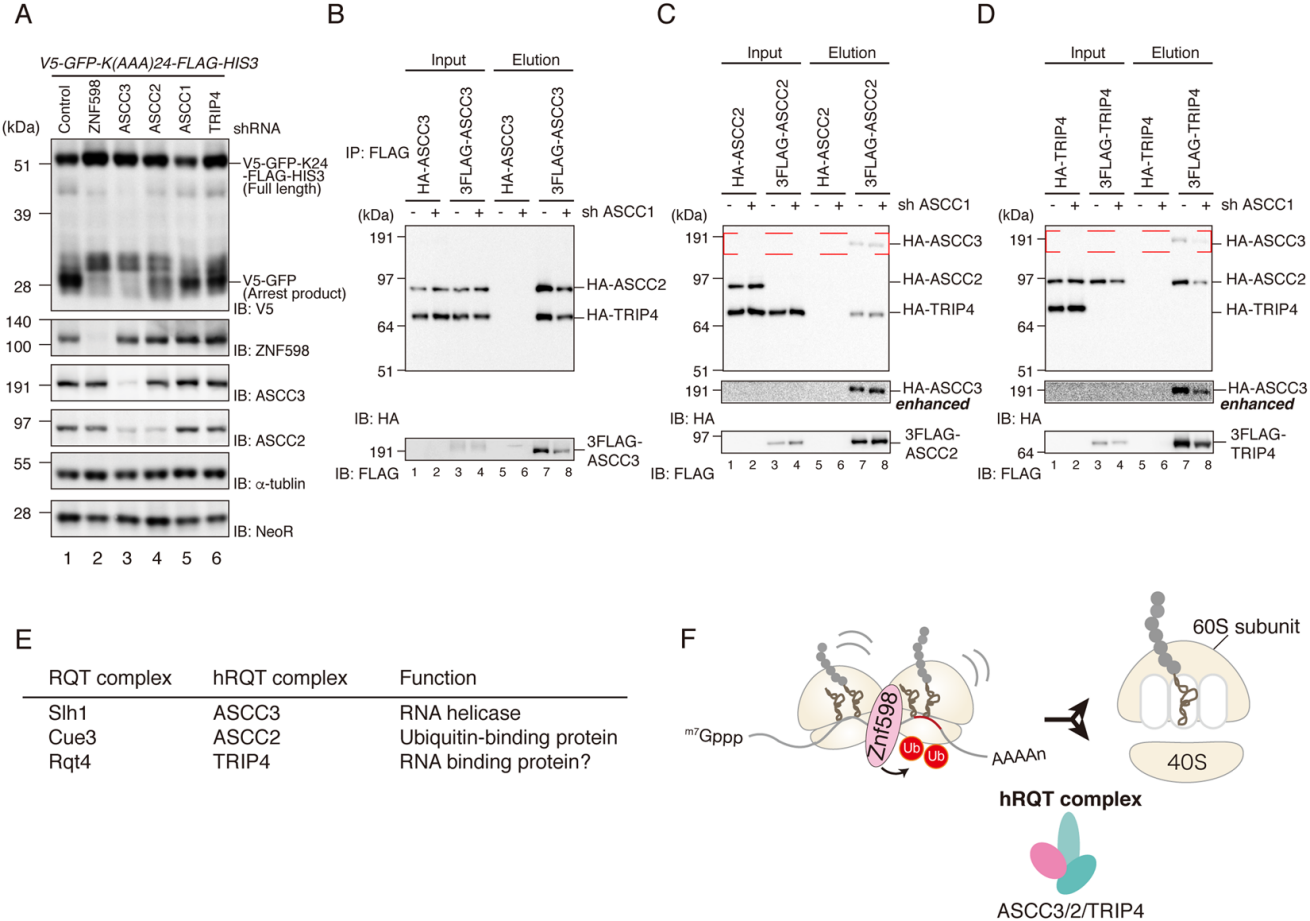
ASCC3/ASCC2-TRIP4 (hRQT complex) is required for RQC. **(A)** The indicated KD cells were transfected with V5-GFP-K(AAA)24-FLAG-HIS3 reporter, and proteins were analyzed by western blotting with anti-V5 antibody. NeoR, expressed from an independent promoter on the reporter plasmid, was used as a control for transfection efficiency. Cropped blots are displayed; full uncropped blots are available in Supplemental Fig. S4. Blots shown are representative of three independent experiments. **(B–D)** FLAG or HA-tagged ASCC3, ASCC2 and TRIP4 were transfected to KD control or ASCC1 KD cells, and total lysates (input) were immunoprecipitated with anti-FLAG antibody beads. FLAG-tagged proteins were eluted with FLAG peptides (elution). Both input and elution fractions were analyzed by western blotting with antibodies against FLAG and HA. HA-ASCC3, highlighted by the red dashed line, is shown with a longer exposure (HA enhanced). Cropped blots are displayed; full uncropped blots are available in Supplemental Fig. S5. Blots shown are representative of three independent experiments. **(E)** Correspondence table of RQT factors in yeast and mammals, along with their functions. **(F)** Proposed model of the hRQT complex, which recognizes ubiquitinated ribosomes and dissociates ribosome into subunits.

After confirming the physical interactions between ASCC3/2/1 and TRIP4, we investigated whether these factors are required for RQC. To this end, we transfected the *V5-GFP-K(AAA)24-FLAG-HIS3* reporter into the corresponding KD cells and monitored RQC induction by western blotting (Fig. 4A). shRNA-mediated KD efficiency is shown in Fig. S1B. In accordance with previous results^8^, ASCC3 KD abolished the induction of RQC. ASCC2 KD and TRIP4 KD partially disrupted the induction of RQC, whereas ASCC1 KD had no effect (Fig. 4A). In ASCC2 or TRIP4 KD cells, the levels of the arrest products were slightly reduced, whereas the levels of the full-length and frameshift products were significantly higher than those in control cells (Fig. 4A). The changes in the full-length products suggest that ribosome stalling was reduced in ASCC2 KD or TRIP4 KD cells, as well as in ZNF598 KD or ASCC3 KD cells. These results indicate that only three members of the ASC-1 complex, ASCC3, ASCC2, and TRIP4, are required for RQC (Fig. 4E,F). We also confirmed that the decrease in the full-length products depends on ribosome stalling induced by the poly(A) sequence (Fig. S1C). Considering that ASCC3, ASCC2, and TRIP4 are orthologs of the yeast RQT complex (Slh1, Cue3, and Rqt4, respectively), we suspected that the hRQT complex was composed of these factors, and that inhibition of hRQT complex-mediated ribosome recognition and/or dissociation may have abolished ribosome stalling and RQC induction. To explore the possibility that the hRQT complex might include novel components, we investigated the interaction of ASCC3/2-TRIP4 in ASCC1 KD cells. As expected, purification of one of these factors resulted in the co-purification of the other two factors, indicating that ASCC3/2-TRIP4 formed a complex even in the absence ASCC1 (Fig. 4B–D). We named the components of this novel hRQT complex hRqt2 (ASCC3), hRqt3 (ASCC2), and hRqt4 (TRIP4).

### ATPase-dependent helicase activity of ASCC3(hRQT2) is required for RQC

**Figure 5:**
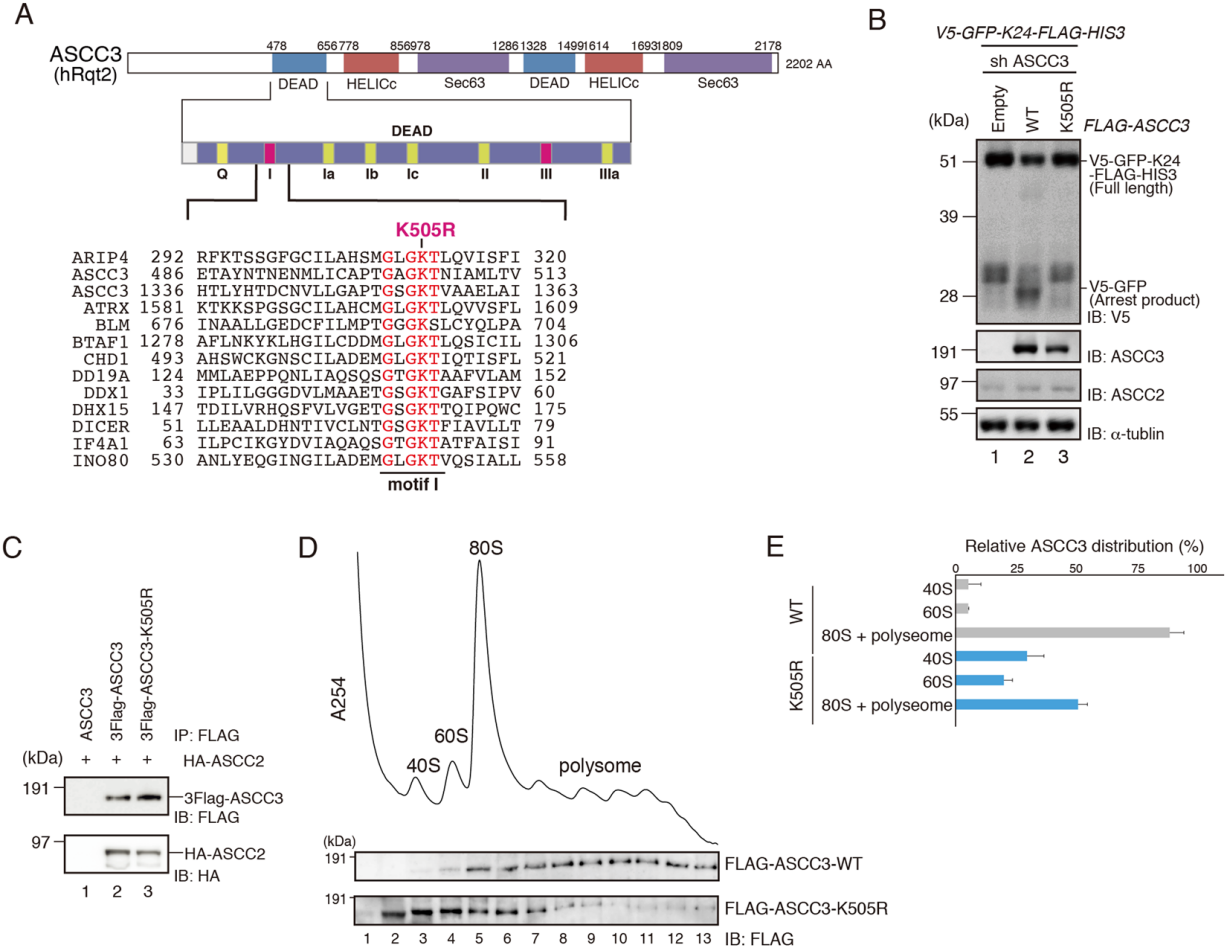
ATPase-dependent helicase activity of ASCC3 is essential for RQC induction. **(A)** Top: Domain structure of ASCC3. Bottom: Amino acid sequence alignment of the conserved residues in the RecA1 motif I of indicated proteins. **(B)** FLAG-ASCC3-WT or K505R mutant was co-transfected into ASCC3 KD cells along with the *V5-GFP-K(AAA)*_24_*-FLAG-HIS3* reporter, and protein was analyzed by western blotting with anti-V5 antibodies. **(C)** HA-ASCC2, and FLAG-ASCC3-WT or K505R mutant were co-transfected to HEK293T cells. Cell lysates were immunoprecipitated with anti-FLAG antibody beads. FLAG-tagged proteins were eluted with FLAG peptides, and the elution fraction was analyzed by western blotting with antibodies against FLAG and HA. **(B**,**C)** Cropped blots are displayed; full uncropped blots are available in Supplemental Fig. S6. Blots shown are representative of three independent experiments. **(D)** FLAG-ASCC3-WT or K505R mutant was transfected into ASCC3 KD cells, and polysome analysis was performed. Top: A_254_ of each fraction is plotted. Bottom: Protein distribution of ASCC3 was analyzed by western blotting with anti-FLAG antibodies. Cropped blots are displayed; full uncropped blots are available in Supplemental Fig. S7. Blots shown are representative of three independent experiments. (**E**) Two independent blots of indicated samples were quantified, and relative ASCC3 distribution is displayed. Error bars represent S.E. from N = 2.

ASCC3(hRQT2), which belongs to the helicase family, harbors two sets of RecA helicase and Sec. 63 domains (Fig. 5A). In yeast, the helicase activity of Cue3(Rqt3) is necessary for RQC before the ribosome dissociation step. To investigate whether the ATPase-dependent helicase activity of ASCC3 is essential for RQC, we mutated the conserved lysine residue in the first RecA domain to arginine residue (K505R), leading to a deficiency in ATPase activity (Fig. 5A). Overexpression of the ASCC3-K505R mutant in ASCC3 KD cells did not complement the disruption of RQC induction (Fig. 5B), although the interaction between the ASCC3-K505R mutant and ASCC2 remained unchanged (Fig. 5C). To see whether ASCC3 WT and ATPase-deficient K505R mutant are associated with ribosome, we performed sucrose density gradient analysis followed by western blotting. In these experiments, we overexpressed ASCC3 WT or K505R mutant in ASCC3 KD cells and analyzed the cell lysates. ASCC3 WT was recruited to the ribosome (Fig. 5D,E), like its yeast ortholog Slh1. The ASCC3 K505R mutant was also recruited to the ribosome, although its distribution was partially shifted to the lighter fraction in comparison with the WT (Fig. 5D,E), indicating that the ribosome binding activity of the ASCC3 K505R mutant was weakened by its deficiency in ATPase activity. These results suggest that as in yeast, the ATPase-dependent helicase activity of ASCC3 is indispensable for RQC in mammalian cells.

### Ubiquitin-binding activity of ASCC2(hRQT3) is required for RQC

**Figure 6:**
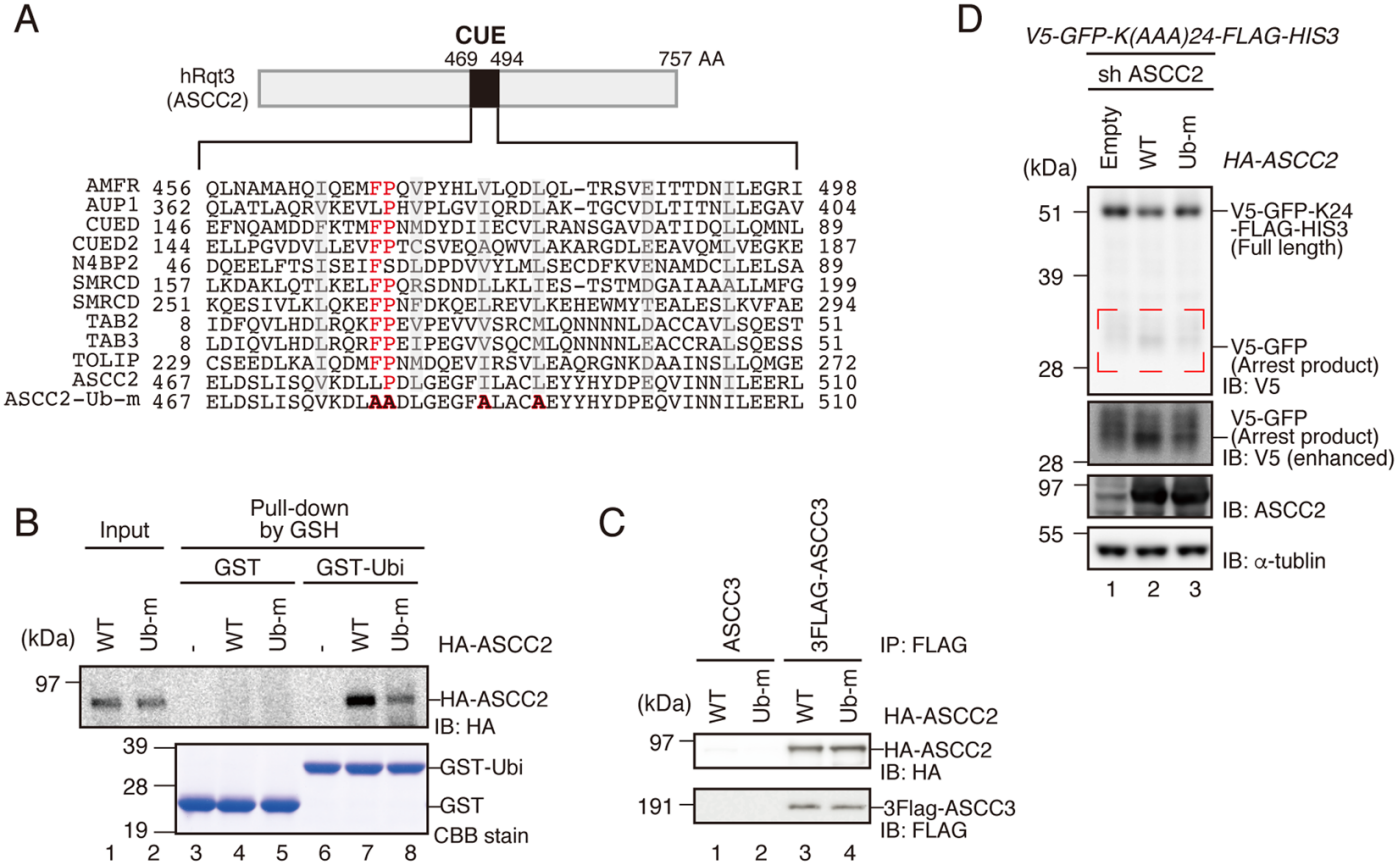
Ubiquitin-binding activity of ASCC2 is crucial for RQC induction. **(A)** Top: Schematic drawing of the domain structure of ASCC2. Bottom: Amino-acid sequence alignment of conserved residues in CUE domains of the indicated proteins. Mutated residues in Ub-m are shown in bold red. **(B)** Recombinant HA-ASCC2 or HA-ASCC2-Ub-m mutant (Input), and GST or GST-Ubiquitin were mixed and reacted *in vitro*, and then pulled down with Glutathione Sepharose. GST-tagged proteins were eluted with glutathione (elution). Both input and elution fractions were analyzed by western blotting with antibodies against HA. Elution fractions were also analyzed by CBB staining. **(C)** HA-ASCC2 or HA-ASCC2-Ub-m mutant was co-transfected into HEK293T cells along with FLAG-ASCC3, and immunoprecipitated with anti-FLAG antibody beads. FLAG-tagged proteins were eluted with FLAG peptides, and the eluted fraction was analyzed by western blotting with antibodies against FLAG and HA. **(D)** HA-ASCC2 or HA-ASCC2-Ub-m mutant was co-transfected into ASCC2 KD cells along with the *V5-GFP-K(AAA)*_24_*-FLAG-HIS3* reporter, and protein was analyzed by western blotting with anti-V5 antibodies. Arrest products highlighted by the red dashed line are shown with a longer exposure (V5 enhanced). **(B–D)** Cropped blots are displayed; full uncropped blots are available in Supplemental Fig. S8. Blots shown are representative of three independent experiments.

ASCC2(hRQT3) prefers to bind to K63-linked poly-ubiquitin chains via its ubiquitin-binding CUE domain, and recognition of the K63-linked poly-ubiquitin chain is required for proper localization of the ASCC-ALKBH3 repairing complex^24^. In yeast, the ubiquitin-binding activity of Cue3(Rqt3) is crucial for the induction of RQC^8^. Given that ubiquitination of eS10 by ZNF598 is a key step for RQC^27^, we hypothesized that the ubiquitin-binding activity of ASCC2 is crucial for RQC. To test this idea, we mutated the conserved ubiquitin-binding domain of ASCC2 (Ub-m) (Fig. 6A) and pulled down with GST-tagged ubiquitin (Fig. 6B). The ubiquitin-binding activity of the recombinant ASCC2 Ub-m mutant protein was lower than that of the ASCC2 WT protein (Fig. 6B), although the interaction with ASCC3 remained unchanged (Fig. 6C). Expression of ASCC2 WT in ASCC2 KD cells slightly decreased the amount of full-length product, whereas the levels of the arrest products were elevated. On the other hand, the expression of Ub-m mutant only partially complemented the phenotype (Fig. 6D). This partial complementation of RQC induction is in accordance with the partial ubiquitin-binding activity of the Ub-m mutant. These results imply that a ubiquitinated stalled ribosome can be recognized by the ASCC2 ubiquitin-binding domain.

## Discussion

RQC is an indispensable quality control system for guaranteeing accurate gene expression, and the detailed process of RQC is highly conserved: ribosome collision caused by translation arrest triggers RQC^8–11^, and the collided ribosomes are ubiquitinated by ZNF598^10^ and dissociated into subunits by an unknown mechanism. In this study, we investigated the dissociation of collided ribosomes and identified a novel hRQT complex consisting of ASCC3(hRQT2), ASCC2(hRQT3), and TRIP4(hRQT4) (Fig. 4F). We showed that the ATPase-dependent helicase activity of ASCC3 was essential for RQC induction (Fig. 5B), and that the ubiquitin-binding activities of ASCC2 and TRIP4 also contributed to RQC, although the requirement was only partial (Figs. 4A and 6D). Hel2, an ortholog of ZNF598 in yeast, poly-ubiquitinates collided ribosomes with K63-linkage^6,8^, and ASCC2 preferentially binds to K63-linked poly-ubiquitin chains^24^. Based on these observations, we propose a model in which ubiquitinated 80S ribosomes are first recognized by ASCC2, and then dissociated into 40S and 60S subunits by ASCC3. We suspect that TRIP4 promotes binding of the hRQT complex to the ribosome via its zinc-finger domain. This model explains the partial RQC phenotypes of ASCC2 KD and TRIP4 KD. In ASCC2 KD, the RNA binding activity of TRIP4 localizes ASCC3 to the ribosome without specificity, resulting in lower efficiency in RQC induction. In TRIP4 KD, ASCC3 is located to ubiquitinated ribosomes by ASCC2, but without TRIP4, it cannot promote dissociation due to unstable association with the ubiquitinated ribosomes, resulting in lower efficiency of RQC induction.

Yeast Slh1(Rqt2) is homologous to the RNA helicase Brr1, a pre-mRNA-splicing factor that plays crucial roles in the regulated remodeling of the spliceosome structure during the splicing reaction^28^. The ATPase activity of Slh1 is crucial for triggering RQC^8^, strongly suggesting that ATPase-dependent RNA helicase activity is required for RQC in mammals. ASCC3 is associated with the alpha-ketoglutarate-dependent dioxygenase AlkB homolog 3 (ALKBH3) and unwinds DNA to generate the single-stranded substrate of the ALKBH3-mediated DNA repair pathway^23^. Our results suggest that the ATPase activity of mammalian ASCC3(hRqt2) is required for RQC (Fig. 5). We propose that the RNA helicase activity of ASCC3(hRqt2) is crucial for the dissociation of the ubiquitinated ribosome into subunits in mammals.

ASCC3 activity is inhibited by its own non-coding short form in the later stages of the response to UV irradiation^29^. UV irradiation slows down transcriptional elongation and induces a shift from the expression of long mRNAs to shorter isoforms. The members of the ASCC complex, ASCC3, ASCC2, and ASCC1, globally suppress nascent transcription in the later stages of the DNA damage response. The expression of the short *ASCC3* RNA isoform is also induced by UV radiation and is required to recover transcription after UV irradiation. The short *ASCC3* RNA isoform functions as a non-coding RNA that counteracts the function of the protein-coding isoform. Based on these, we speculate that RQC is regulated by UV irradiation.

We demonstrated that ASCC1 is not required for RQC induction and that the novel hRQT complex, composed of ASCC3/ASCC2/TRIP4, can form a complex without ASCC1 (Fig. 4B–D). On the other hand, ASCC1 coordinates the proper recruitment of the ASCC3 to ASCC2-positive foci via its RNA ligase-like domain during DNA alkylation damage^25^, and ASCC3/ASCC2/ASCC1 (i.e., the ASCC complex) and ASCC3/ASCC2/ASCC1/TRIP4 (i.e., the ASC-1 complex) play roles in DNA alkylation damage repair and transcriptional regulation, respectively^21–23^. The main difference between these events is localization: RQC is a cytosolic event, whereas the others are nuclear event. According to the Human Protein Atlas^30^, all four factors localize in both the cytosol and nucleus, so we suspect that localization of factors are regulated depending on the cellular environment, such as translation stalling, alkylation damage, and serum depletion. Further studies should seek to reveal the sophisticated complex formation process in response to changes in circumstances.

Our identification of the mammalian RQT complex in this study emphasizes the universality of RQC. Our findings imply the existence of other translation-coupled quality control systems in mammals, including the mRNA surveillance system called No-go decay (NGD) and the ribosome surveillance system called Non-functional ribosome decay (NRD). In recent years, accumulating evidence has shown that ribosome ubiquitination monitors cellular processes^6,31^, a phenomenon referred to as the “ribosome ubiquitin code”. Moreover, drugs that specifically block translation of individual transcripts are under development^32,33^. A deeper understanding of translation-coupled quality control mechanisms would open up new paths to drug development.

## Materials and Methods

### Plasmid construction

Plasmids used in this study are listed in Table 1. Plasmids were constructed by PCR-based methods and cloned into pcDNA3.1(+) or pFUGW vector. PrimeSTAR HS DNA polymerase (#BIO-21040/DM5, Takara Bio, Kusatsu, Shiga, Japan) was used for amplification, and T4 DNA ligase (# M0202L, NEB, Ipswich, MA, USA) was used to insert DNA fragments into vectors.

**Table 1:** Plasmids used in this study.

Name	Features	Sourse
pcDNA3.1(+)-*V5-GFP-FLAG-HIS3*	V5-GFP-FLAG-HIS3 reporter	*Matsuo and Ikeuchi et al*., 2018
pcDNA3.1(+)-*V5-GFP-K(AAA)24-FLAG-HIS3*	V5-GFP-K(AAA)24-FLAG-HIS3 reporter	*Matsuo and Ikeuchi et al*., 2018
pcDNA3.1(+)*-3xFLAG-ASCC3*	ASCC3 transcript variant 1 expression vector (NM_006828.4)	This study
pcDNA3.1(+)*-HA-ASCC3*	ASCC3 transcript variant 1 expression vector (NM_006828.4)	This study
pcDNA3.1(+)-*3xFLAG-ASCC2*	ASCC2 transcript variant 4 expression vector (NM_001369921.1)	This study
pcDNA3.1(+)-*HA-ASCC2*	ASCC2 transcript variant 4 expression vector (NM_001369921.1)	This study
pcDNA3.1(+)-*3xFLAG-ASCC1*	ASCC1 transcript variant 2 expression vector (NM_001198800.3)	This study
pcDNA3.1(+)-*HA-ASCC1*	ASCC1 transcript variant 2 expression vector (NM_001198800.3)	This study
pcDNA3.1(+)-*3xFLAG-TRIP4*	TRIP4 transcript variant 2 expression vector (NM_001321924.2)	This study
pcDNA3.1(+)-*HA-TRIP4*	TRIP4 transcript variant 2 expression vector (NM_001321924.2)	This study
pFUGW*-FLAG-ASCC3 shR*	shRNA resistant ASCC3 expression vector	This study
pFUGW*-FLAG-ASCC3 shR K505R*	shRNA resistant ASCC3 K505R expression vector	This study
pcDNA3.1(+)-*HA-ASCC2 shR*	shRNA resistant ASCC2 expression vector	This study
pcDNA3.1(+)*-HA-ASCC2 shR Ub-m*	shRNA resistant ASCC2 Ub-m expression vector	This study
pGEX4T2-*GST*	recombinant protein of GST	This study
pGEX4T2-*GST-Ubiquitin*	recombinant protein of GST-Ubiquitin	This study
pGEX6P2-*GST-prescission site-HA-ASCC2*	recombinant protein of HA-ASCC2	This study
pGEX6P2-*GST-prescission site-HA-ASCC2-Ub-m*	recombinant protein of HA-ASCC2-Ub-m	This study
pcDNA3.1(+)-HA-ZNF598(1-904) shR	shRNA resistant ZNF598 expression vector (NM_178167.4)	This study
pcDNA3.1(+)-HA-ZNF598(ΔRING) shR	shRNA resistant ZNF598 ΔRING expression vector	This study
pcDNA3.1(+)-HA-ZNF598(C29SC32S) shR	shRNA resistant ZNF598 C29SC32S expression vector	This study
pcDNA3.1(+)-HA-ZNF598(1-634) shR	shRNA resistant ZNF598 1-634 expression vector	This study
pcDNA3.1(+)-HA-ZNF598(1-278) shR	shRNA resistant ZNF598 1-278 expression vector	This study
pcDNA3.1(+)-HA-ZNF598(1-246) shR	shRNA resistant ZNF598 1-246 expression vector	This study
pcDNA3.1(+)-HA-ZNF598(1-186) shR	shRNA resistant ZNF598 1-186 expression vector	This study
pcDNA3.1(+)-HA-ZNF598(21-904) shR	shRNA resistant ZNF598 21-904 expression vector	This study
pcDNA3.1(+)-HA-ZNF598(21-278) shR	shRNA resistant ZNF598 21-278 expression vector	This study
pcDNA3.1(+)-Rluc-K24-Fluc	Rluc-K24-Fluc reporter	This study

### Cell culture and transfection

HEK293T cells were cultured in DMEM medium (Nacalai Tesque, Kyoto, Kyoto, Japan) supplemented with 10% Fetal Bovine Serum (Sigma-Aldrich, St. Louis, MO, USA) and Penicillin-Streptomycin (Fujifilm Wako pure chemical corporation, Osaka, Osaka, Japan). Plasmid transfection was performed using the PEI-MAX reagent (Cosmo Bio, Koto, Tokyo, Japan).

### Construction of knockdown cells

All knockdown cells were generated using shRNA-expressing lentivirus^8^. shRNAs against respective target genes were cloned into the *Hpa*I/*Xho*I restriction sites of pLL3.7, in which the GFP gene was replaced with a puromycin resistant gene. HEK293T cells infected with shRNA-expressing lentivirus were selected with 10 µg/ml puromycin. shRNA target sequences are listed in Table 2.

**Table 2:** Target sequence of shRNAs.

**Name**	**Targeting sequences**
non silencing	5′-TCCTAAGGTTAAGTCGCCCTCGCTCGAGCGAGGGCGACTTAACCTTAGGTTTTTG-3′
shZNF598-1	5′-GCCAGTTGCCGTCGTCGTTAAT-3′
shASCC3-2	5′-GTAATGCTACTAATCGAATTA-3′
shASCC2-5	5′-GAGCAGGTGATCAACAATAT-3′
shASCC1-2	5′-GCATCGAAATGGTGTAATTT-3′
shASCC1-4	5′-GCATGGTGGATGTTCTTTA-3′
shTRIP4-2	5′-GAATGATCAGGAGTTGATTT-3′
shTRIP4-4	5′-GATCCTGGAAGAAGAAAATT-3′

### Neutral PAGE and western blotting

Samples for Neutral PAGE were prepared as follows. Cultured cells were lysed using a passive lysis buffer (Promega, Madison, WI, USA). Lysed cells were collected and centrifuged at 13,5000 *g* for 1 min at 4 °C. Supernatants were transferred to another tube, and protein concentrations were measured by Bradford method (Bio-Rad, Hercules, CA, USA). The samples were adjusted to contain the same amounts of total protein, mixed with NuPAGE sample buffer (200 mM Tris-HCl pH 6.8, 8% w/v SDS, 40% glycerol, 0.04% BPB, and 100 mM DTT), and heated at 65 °C for 10 min. Protein samples were electrophoresed for 100 min at 50 mA constant current in MOP-SDS buffer (1 M MOPS, 1 M Tris base, 69.3 mM SDS, and 20.5 mM EDTA), using 10% polyacrylamide gel at neutral pH (pH 6.8). After electrophoresis, samples were transferred to PVDF membrane (IPVH00010, Millipore, Burlington, MA, USA). Membranes were blocked with PBS containing 0.1% Tween-20 (Sigma-Aldrich, St. Louis, MO, USA) and 5% (w/v) skim milk. Blots were probed with the primary antibodies (Table 3) and corresponding secondary antibodies (Table 3). The membrane was incubated with ECL solution (Perkin Elmer, Waltham, MA, USA), and then chemiluminescence was collected on an Image Quant LAS4000 (GE Healthcare, Chicago, IL, USA)^34^.

**Table 3:** Antibodies used in this study.

Anti-body Name	SOURCE	IDENTIFIER	Dilution
anti-FLAG M2 antibody	Sigma-Aldrich	F1804-1MG	1:5000
anti-HA-Peroxidase	Roche	12013819001	1:3000
Mouse monoclonal anti-V5-Tag	BIO-RAD	MCA1360	1:3000
Rabbit polyclonal anti-ZNF598	Novus Biologicals	NBP1-84658	1:2000
Rabbit polyclonal anti-ASCC3	Proteintech	17627-1-AP	1:2000
Rabbit polyclonal anti-ASCC2	BETHYL	A304-020A	1:2000
Mouse monoclonal anti-α-tubulin	Sigma-Aldrich	T6074	1:3000
﻿Rabbit polyclonal anti-neomycin phosphotransferase II	Merck KGaA	06-747	1:2000
HRP-Linked anti-rabbit IgG antibodies	GE Healthcare	NA934	1:5000
HRP-Linked anti- mouse IgG antibodies	GE Healthcare	NA931	1:5000

### RNA extraction and RT-qPCR

RNA was extracted from cells using ISOGEN II (NIPPON GENE, Chiyoda, Tokyo, Japan). For each sample, 500 ng RNA was subjected to reverse transcription using ReverTra Ace (TOYOBO, Osaka, Osaka, Japan) with an oligo dT primer (5′-TTTTTTTTTTTTTTT-3′). Reverse-transcribed products (cDNA) were subjected to quantification using the KAPA SYBR FAST Universal qPCR kit (KAPA Biosystems, Wilmington, MA, USA) on a CFX Connect Real-Time System (Bio-Rad, Hercules, CA, USA)^34^. qPCR primers are listed in Table 4.

**Table 4:** Primers used in this study.

Name	description	sequences	plasmid
OIT9556	KpnI-3×FLAG-ASCC3 Fw	5′-AAGCTTGGTACCATGGACTACAAGGACGACGATGACAAGGACTACAAGGACGACGATGACAAGGACTACAAGGACGACGATGACAAGGCTTTACCTCGTCTC-3′	pcDNA3.1(+)*-3xFLAG-ASCC3*
OIT9557	KpnI-HA-ASCC3 Fw	5′-AAGCTTGGTACCATGTACCCATACGATGTTCCAGATTACGCTGCTTTACCTCGTCTC-3′	pcDNA3.1(+)*-HA-ASCC3*
OIT9473	ASCC3-BamHI Rev	5′-ACTAGTGGATCCTTACTTTAATGCCAG-3′	pcDNA3.1(+)-3xFLAG-ASCC3, pcDNA3.1(+)-HA-ASCC3
OIT9474	BamHI-3×FLAG-ASCC2 Fw	5′-GAGCTCGGATCCATGGACTACAAGGACGACGATGACAAGGACTACAAGGACGACGATGACAAGGACTACAAGGACGACGATGACAAGCCAGCTCTGCCCCTG-3′	pcDNA3.1(+)-*3xFLAG-ASCC2*
OIT9475	BamHI-HA-ASCC2 Fw	5′-GAGCTCGGATCCATGTACCCATACGATGTTCCAGATTACGCTCCAGCTCTGCCCCTG-3′	pcDNA3.1(+)-*HA-ASCC2*
OIT9476	ASCC2-EcoRI Rev	5′-TCTGCAGAATTCTCAGGATGGGATCATG-3′	pcDNA3.1(+)-*3xFLAG-ASCC2*, pcDNA3.1(+)-HA-ASCC2
OIT9477	KpnI-3×FLAG-ASCC1 Fw	5′-AAGCTTGGTACCATGGACTACAAGGACGACGATGACAAGGACTACAAGGACGACGATGACAAGGACTACAAGGACGACGATGACAAGGAAGTTCTGCGTCC-3′	pcDNA3.1(+)-*3xFLAG-ASCC1*
OIT9478	KpnI-HA-ASCC1 Fw	5′-AAGCTTGGTACCATGTACCCATACGATGTTCCAGATTACGCTGAAGTTCTGCGTCC-3′	pcDNA3.1(+)-*HA-ASCC1*
OIT9479	ASCC1-BamHI Rev	5′-ACTAGTGGATCCTCAGGAGAAGTCAAT-3′	pcDNA3.1(+)-*3xFLAG-ASCC1*
OIT9937	KpnI-3xFLAG-TRIP4 isoform1 Fw	5′-AAGCTTGGTACCATGGACTACAAGGACGACGATGACAAGGACTACAAGGACGACGATGACAAGGACTACAAGGACGACGATGACAAGGCGGTGGCTGGGGCG-3′	pcDNA3.1(+)-*3xFLAG-TRIP4*
OIT9936	KpnI-HA-TRIP4 isoform1 Fw	5′-AAGCTTGGTACCATGTACCCATACGATGTTCCAGATTACGCTGCGGTGGCTGGGGCG-3′	pcDNA3.1(+)-*HA-TRIP4*
OIT9482	TRIP4-BamHI Rev	5′-ACTAGTGGATCCTCAGACAGCTTTATTC-3′	pcDNA3.1(+)-*3xFLAG-TRIP4*, pcDNA3.1(+)-HA-TRIP4
OIT6992	ASCC3 shR Fw	5′-CTGCCACTGCAGCTTGTAATGCAACTAACCGAATCATTTCTCATTTTAGTC-3′	pFUGW*-FLAG-ASCC3 shR*
OIT6993	ASCC3 shR Rev	5′-GACTAAAATGAGAAATGATTCGGTTAGTTGCATTACAAGCTGCAGTGGCAG-3′	pFUGW*-FLAG-ASCC3 shR*
OIT9845	ASCC3 505R Fw	5′-CCTACAGGAGCTGGACGCACCAACATTGCAATG-3′	pFUGW*-FLAG-ASCC3 shR K505R*
OIT9846	ASCC3 505R Rev	5′-CATTGCAATGTTGGTGCGTCCAGCTCCTGTAGG-3′	pFUGW*-FLAG-ASCC3 shR K505R*
OIT9849	ASCC2 shR Fw	5′-CTACGACCCAGAGCAAGTGATTAACAACATCCTGGAGGAGCGG-3′	pcDNA3.1(+)-*HA-ASCC2 shR*
OIT9850	ASCC2 shR Rev	5′-CCGCTCCTCCAGGATGTTGTTAATCACTTGCTCTGGGTCGTAG-3′	pcDNA3.1(+)-*HA-ASCC2 shR*
OIT9847	ASCC2 Ub-m Fw	5′-CAAGTGAAGGACCTGGCGGCCGA“CCTTGGTGAGGGCTTCGCCCTGGCCTGCGCGGAGTACTACCACTAC-3′	pcDNA3.1(+)*-HA-ASCC2 shR Ub-m*
OIT9848	ASCC2 Ub-m Rev	5′-GTAGTGGTAGTACTCCGCGCAGGCCAGGGCGAAGCCCTCACCAAGGTCGGCCGCCAGGTCCTTCACTTG-3′	pcDNA3.1(+)*-HA-ASCC2 shR Ub-m*
OIT6546	ZNF598sh1R_F1	TTGAACGGTACCATGTACCCATACGACGTCCCAGACTACGCGATGGCGGCGGCGGG	pcDNA3.1(+)-HA-ZNF598(1-904) shR
OIT6490	ZNF598sh1R_F2	CAGCAGAGCCGAGGGGCCAGTTGCTGTCGTTGTTAACGGACACACGGAGGGC	pcDNA3.1(+)-HA-ZNF598(1-904) shR
OIT6489	ZNF598sh1R_R1	GCCCTCCGTGTGTCCGTTAACAACGACAGCAACTGGCCCCTCGGCTCTGCTG	pcDNA3.1(+)-HA-ZNF598(1-904) shR
OIT6547	ZNF598sh1R_R2	TAGATGGAATTCCTACGTGATGATCCTGGCGATGGCTTGCAGGGAGGGGAAGTCGTCGTCCCGGGC	pcDNA3.1(+)-HA-ZNF598(1-904) shR
OIT6897	KpnI_HA-ZNF598_ΔRING_sh1R_FW	TTGAACGGTACCATGTACCCATACGACGTCCCAGACTACGCGATGGCGGCGGCGGGGGGCGCCGAGGGGCGGCGCGCGGCCCTGGAGGCGGCGGCGGCGGCAGCTCCTGAGCGGGGAGGCGGGAGCGAGGAGCTGCGCCAGGTGGT	pcDNA3.1(+)-HA-ZNF598(ΔRING) shR
OIT6898	HA-ZNF598_ΔRING_sh1R_SacII_Rev	TAGATGCCGCGGGCACTCGTGCTGCAGCAG	pcDNA3.1(+)-HA-ZNF598(ΔRING) shR, pcDNA3.1(+)-HA-ZNF598(C29SC32S) shR
OIT6899	KpnI_HA-ZNF598_C29SC32S_sh1R_FW	TTGAACGGTACCATGTACCCATACGACGTCCCAGACTACGCGATGGCGGCGGCGGGGGGCGCCGAGGGGCGGCGCGCGGCCCTGGAGGCGGCGGCGGCGGCAGCTCCTGAGCGGGGAGGCGGGAGCTCAGTGCTGTCATGCGGAGACCTG	pcDNA3.1(+)-HA-ZNF598(C29SC32S) shR
OIT6900	SacII_HA-ZNF598_1-1902_sh1R_FW	TAGATGCCGCGGTGCCCCGAGCTGCCACCTTTCAGC	pcDNA3.1(+)-HA-ZNF598(1-634) shR, pcDNA3.1(+)-HA-ZNF598(1-246) shR
OIT6901	HA-ZNF598_1-1902_sh1R_EcoRI_Rev	TTGAACGAATTCCTAGTTAACAACGACAGCAACTGGCCCCTCGGCTCTGCTGGCAG	pcDNA3.1(+)-HA-ZNF598(1-634) shR
OIT5292	KpnI_HA-ZNF598_d559-2715FW	TTGAACGGTACCATGTACCCATACGACGTCCCAGACTACG	pcDNA3.1(+)-HA-ZNF598(1-278) shR, pcDNA3.1(+)-HA-ZNF598(1-186) shR
OIT5294	EcoRI_HA-ZNF598_835-2715_Rev	TTGAACGAATTCCTAGCGTGCCTCGGCGCGGCTG	pcDNA3.1(+)-HA-ZNF598(1-278) shR, pcDNA3.1(+)-HA-ZNF598(21-278) shR
OIT6902	HA-ZNF598_1-738_sh1R_EcoRI_Rev	TAGATGGAATTCCTAGCGGCCTTCCTCACACAGAAAGTGCTTCTCCCGGAAGTGCTCA	pcDNA3.1(+)-HA-ZNF598(1-246) shR
OIT5293	EcoRI_HA-ZNF598_d559-2715Rev	TAGATGGAATTCCTAGTGCCCACGGTGCGACGTGTCA	pcDNA3.1(+)-HA-ZNF598(1-186) shR
OIT6904	KpnI_HA-ZNF598_61-2715_sh1R_FW	TAGATGGGTACCATGTACCCATACGACGTCCCAGACTACGCGATGG	pcDNA3.1(+)-HA-ZNF598(21-904) shR
OIT6905	HA-ZNF598_61-2715_sh1R_SacII_Rev	TTGAACCCGCGGGCACTCGTGCTGCAGCAG	pcDNA3.1(+)-HA-ZNF598(21-904) shR
OIT5295	KpnI_HA-ZNF598_d1-60_835-2715_FW	TAGATGGGTACCATGTACCCATACGACGTCCCAGACTACGC	pcDNA3.1(+)-HA-ZNF598(21-278) shR
OIT3190	Rluc fwd	tagatggctagcgccgccaccatgacttcgaaagtttatgatccagaacaa	pcDNA3.1(+)-Rluc-K24-Fluc
OIT3195	Fluc rev	ttgaacCTCGAGTCACAATTTGGACTTTCCGCCCTTCTTGGC	pcDNA3.1(+)-Rluc-K24-Fluc
OIT 3846	K24_sense	AGCTTaaaaaaaaaaaaaaaaaaaaaaaaaaaaaaaaaaaaaaaaaaaaaaaaaaaaaaaaaaaaaaaaaaaaaaaaGC	pcDNA3.1(+)-Rluc-K24-Fluc
OIT 3847	K24_antisense	GGCCGCttttttttttttttttttttttttttttttttttttttttttttttttttttttttttttttttttttttttA	pcDNA3.1(+)-Rluc-K24-Fluc
OIT5476	hZnf598_qPCR_Fw	5′-GGAACGAGGGGGTCGTTG-3′	qPCR
OIT5477	hZnf598_qPCR_Rev	5′-TTGTACCTCCAGCTTCCTCG-3′	qPCR
OIT6136	hASCC3_qPCR_F	5′-ATCAAATTGCATGCTGACCA-3′	qPCR
OIT6137	hASCC3_qPCR_R	5′-TGATTTGGGAAATCGAGGAG-3′	qPCR
OIT6140	hASCC2_qPCR_F	5′-TTCCACATCATCCTGAACCA-3′	qPCR
OIT6141	hASCC2_qPCR_R	5′-TAGTCCCGGAGGAACCTCTT-3′	qPCR
OIT6144	hASCC1_qPCR_F	5′-GAAAGCGCCCTTCACTCAC-3′	qPCR
OIT6145	hASCC1_qPCR_R	5′-TGGAAAATGCTGCTGTCAAC-3′	qPCR
OIT 6253	hGAPDH_qPCR-4_Fw	5′-AGGGCTGCTTTTAACTCTGGT-3′	qPCR
OIT 6254	hGAPDH_qPCR-4_Rev	5′-CCCCACTTGATTTTGGAGGGA-3′	qPCR
OIT10032	hTRIP4-qPCR-F	5′-CGAGAGGAGGAGCTGAGAGA-3′	qPCR
OIT10033	hTRIP4-qPCR-R	5′-GGCAATGGCCTGTATTGTCT-3′	qPCR

### Polysome analysis

Before harvest, cells were treated with 100 μg/mL cycloheximide (#06741–04, Nacalai Tesque, Kyoto, Kyoto, Japan) for 10 min at 37 °C. Then, the cells were harvested in ice-cold PBS (#07269–84, Nacalai Tesque, Kyoto, Kyoto, Japan) containing 100 μg/mL cycloheximide. Harvested cells were washed with ice-cold PBS containing 100 μg/mL cycloheximide resuspended in the lysis buffer (10 mM HEPES-KOH pH 7.4, 100 mM NaCl, 10 mM MgCl_2_, 2 v/v% NP-40, 2 mM DTT, 1 mM PMSF, 100 μg/mL CHX, cOmplete Mini EDTA-free Protease Inhibitor Cocktail). To prepare crude lysate, the cell suspension was incubated on ice for 10 min, and the insoluble fraction was removed by centrifugation at 7,300 g, 4 °C for 15 min. Sucrose gradients (10–50% sucrose in 20 mM HEPES-KOH pH 7.4, 100 mM KCl, 5 mM MgCl_2_, 1 mM DTT, 100 μg/mL CHX) were prepared in 14 × 89 mm open-top polyclear centrifuge tubes (#7030, Seton Scientific, Petaluma, CA, USA) using a Gradient Master (Cosmo Bio, Koto, Tokyo, Japan). The lysates (equivalent of 50 A260 units) were placed on top of the gradients and then centrifuged at 280,000 g in a SW41Ti rotor (Beckman Coulter, Brea, CA, USA) for 1.5 h at 4 °C. The gradients were fractionated and polysome profiles were drawn as previously described^8^. Equal amounts of each fraction were subjected to western blot analysis, and the blots were quantified using Multi Gauge V.3.0 (Fujifilm, Minato, Tokyo, Japan).

### Immunoprecipitation of RQT factors

HEK293T cells overexpressing individual RQT factors under the control of the CMV promoter were grown exponentially at 37 °C. After washing with ice-cold PBS, cell were was resuspended by passage through a 25 G syringe in ice-cold LB100 (50 mM Tris-HCl pH 7.5, 100 mM NaCl, 10 mM MgCl_2_, 10% Glycerol, 10 µM ZnCl_2,_ 10 mM L-Arginine, 2% NP-40, 5 mM DTT, 1 mM PMSF) containing cOmplete Mini EDTA-free Protease Inhibitor Cocktail (#11836170001, Roche, Basel, Basel-Stadt, Switzerland) (1 tablet/10 mL) (#N-2525R, Fujifilm Wako pure chemical corporation, Osaka, Osaka, Japan), and then centrifuged at 7,300 g, at 4 °C for 10 min; centrifugation was performed three times to obtain a clear lysate. To purify complex containing FLAG-tagged proteins, 30 µL of pre-equilibrated anti-DYKDDDDK tag antibody beads (#016–22784, Fujifilm Wako pure chemical corporation, Osaka, Osaka, Japan) was incubated and bound to FLAG tag in the obtained lysate, and then eluted with 450 µL of LB100 containing 250 µg/mL FLAG peptide (GenScript Biotech, Piscataway, NJ, USA) for 3 h. The purified complexes were concentrated by TCA precipitation and dissolved in SDS sample buffer^8^.

### Recombinant protein purification

Recombinant GST and GST-Ubiquitin were cloned in pGEX4T2 vector. In this pull-down analysis, GST-fusion proteins were eluted by Glutathione-Reduced Form (GSH) (#073-02013, Fujifilm Wako pure chemical corporation, Osaka, Osaka, Japan). These recombinant proteins are not eluted by Prescission protease, since these GST-fusion proteins derived from pGEX4T2 vector do not contain Prescission protease sites. On the other hand, recombinant HA-ASCC2 or HA-ASCC2 Ub-m were cloned in pGEX6P2 vector and GST-fusion protein derived from pGEX6P2 vector contain Prescission protease sites. Each recombinant protein was purified with Glutathione Sepharose 4B (#17-1756-05, GE Healthcare, Chicago, IL, USA) from *E. coli* Rossetta-gami 2 cells as described in a previous study^6,31^. The proteins were eluted from beads using 500 µL Elution buffer (12 µL Prescission protease or 10 mM GSH in GB100 adjusted to pH 8.0) at 4 °C for 16 h^8,31^.

### Ubiquitin-binding assay

For ubiquitin-binding assays, 20 µg GST and GST-Ub were incubated with 10 µL Glutathione Sepharose 4B in a total volume of 200 µl GB100 buffer at 4 °C for 1 h, followed by three washes with GB100 buffer. Glutathione Sepharose 4B-bound GST- or GST-Ub was incubated overnight with rotation at 4 °C with 100 ng HA-ASCC2 or HA-ASCC2 Ub-m in 500 µL GB buffer. The GST or GST-Ub bound fractions were eluted with 400 μL GSH, concentrated by TCA precipitation, dissolved in 30 μL Laemmli buffer, analyzed by NuPAGE, and stained with Coomassie blue or subjected to western blot analysis as described above.

## Supplementary information

Supplementary Information.
