# Supplementary material for: Identification of a novel trigger complex that facilitates ribosome-associated quality control in mammalian cells
Source: Sci Rep. 2020 Feb 25;10:3422. doi: 10.1038/s41598-020-60241-w (PMC7042231; doi:10.1038/s41598-020-60241-w)
Supplement: Supplementary file 1 — Supplementary Information. [file 41598_2020_60241_MOESM1_ESM.pdf]

Supplementary material

Identification of a novel trigger complex that facilitates ribosome-associated quality control in mammalian cells

Authors: Satoshi Hashimoto[1,2], Takato Sugiyama[1,2], Reina Yamazaki[1], Risa Nobuta[1] and Toshifumi Inada[1]*

Affiliations:

[1]From the Graduate School of Pharmaceutical Sciences, Tohoku University, Sendai 980-8578, Japan.

[2]These authors contributed equally to this study.

This PDF file includes:

Figure S1-S9

Fig. S1

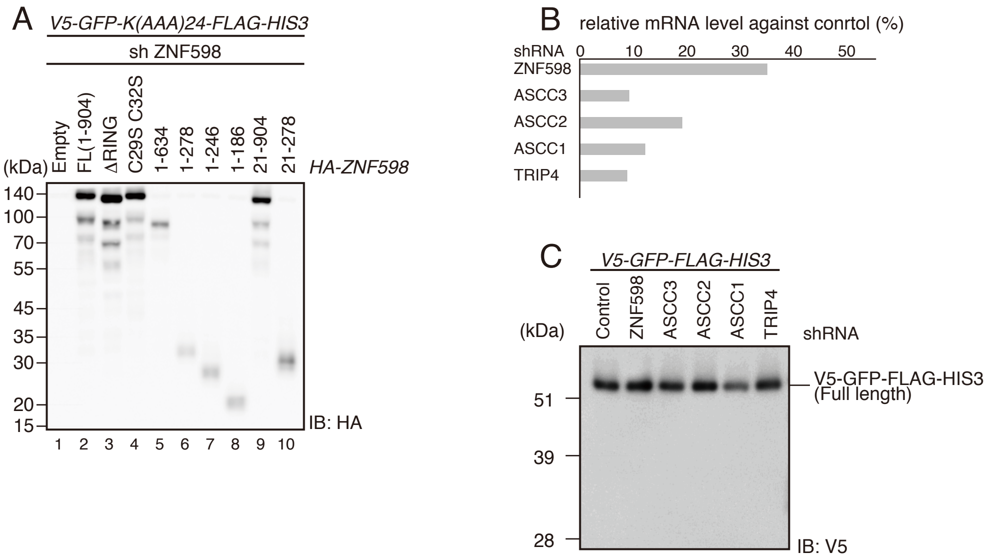
A
V5-GFP-K(AAA)24-FLAG-HIS3
sh ZNF598
Empty
FL(1-904)
ΔRING
C29S C32S
1-634
1-278
1-246
1-186
21-904
21-278
HA-ZNF598
(kDa)
140
100
70
55
45
35
30
20
15
IB: HA
1 2 3 4 5 6 7 8 9 10
B
relative mRNA level against conrtol (%)
shRNA
0 10 20 30 40 50
ZNF598
ASCC3
ASCC2
ASCC1
TRIP4
C
V5-GFP-FLAG-HIS3
Control
ZNF598
ASCC3
ASCC2
ASCC1
TRIP4
shRNA
(kDa)
51
39
28
V5-GFP-FLAG-HIS3
(Full length)
IB: V5

Fig. S2

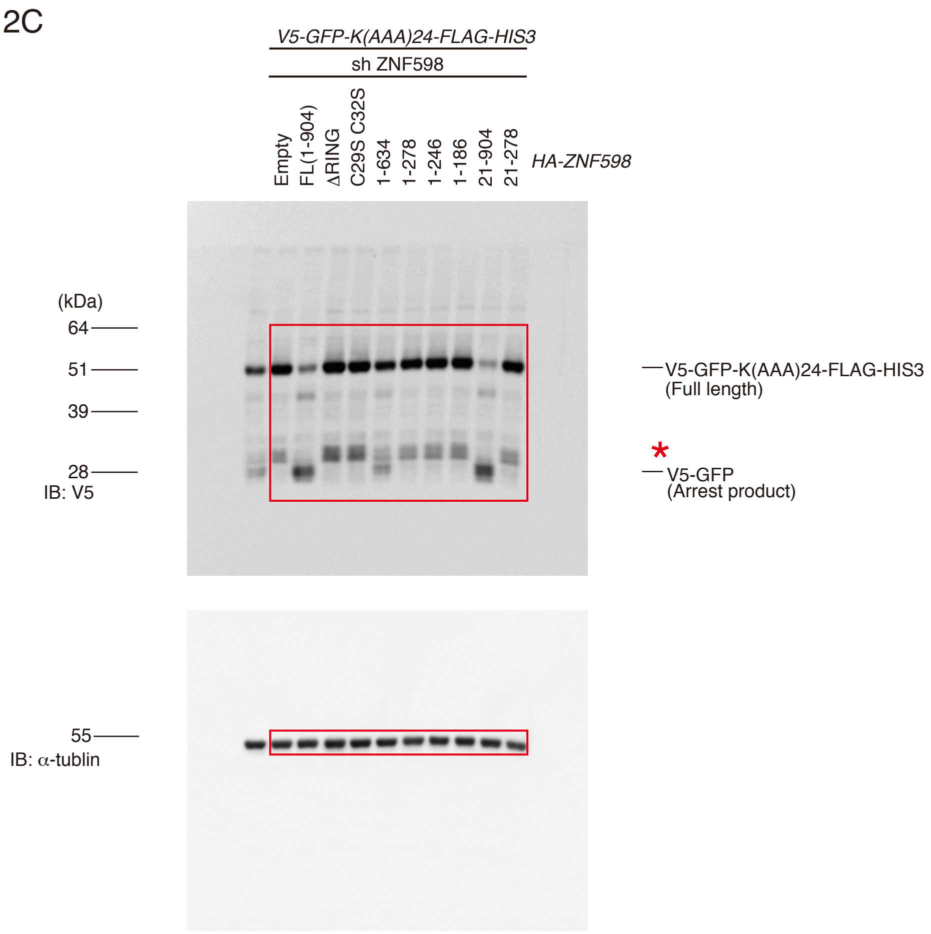
2C
V5-GFP-K(AAA)24-FLAG-HIS3
sh ZNF598
Empty
FL(1-904)
ΔRING
C29S C32S
1-634
1-278
1-246
1-186
21-904
21-278
HA-ZNF598
(kDa)
64
51
39
28
IB: V5
V5-GFP-K(AAA)24-FLAG-HIS3
(Full length)
*
V5-GFP
(Arrest product)
55
IB: α-tublin

Fig. S3

3B-E

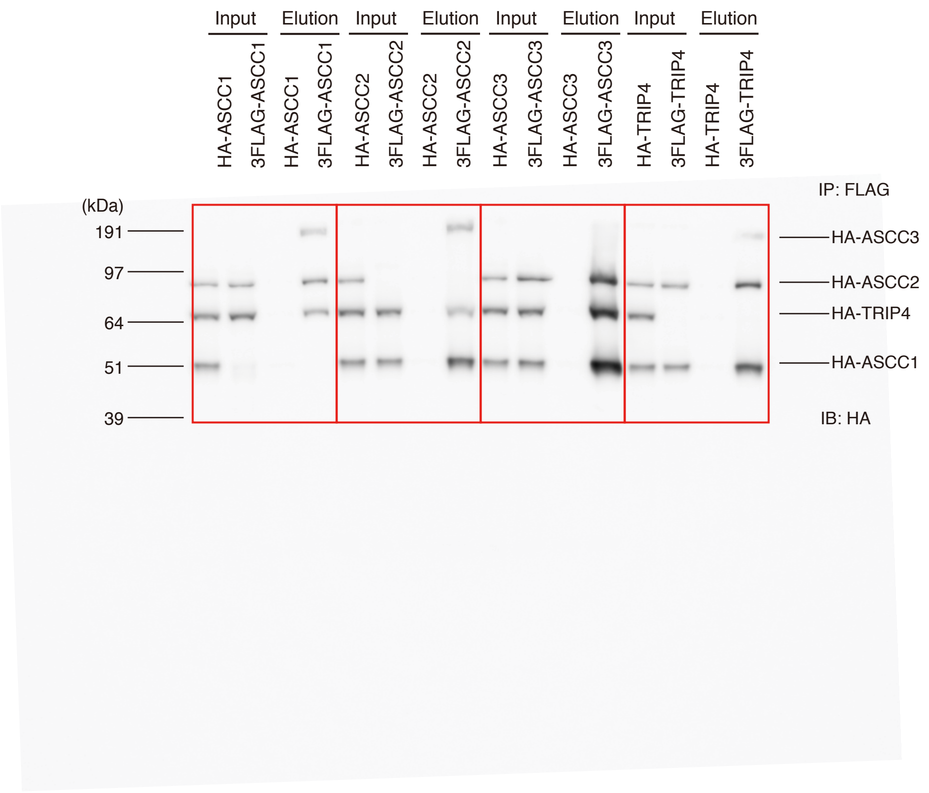
Input
Elution
Input
Elution
Input
Elution
Input
Elution
HA-ASCC1
3FLAG-ASCC1
HA-ASCC1
3FLAG-ASCC1
HA-ASCC2
3FLAG-ASCC2
HA-ASCC2
3FLAG-ASCC2
HA-ASCC3
3FLAG-ASCC3
HA-ASCC3
3FLAG-ASCC3
HA-TRIP4
3FLAG-TRIP4
HA-TRIP4
3FLAG-TRIP4
IP: FLAG
(kDa)
191
97
64
51
39
HA-ASCC3
HA-ASCC2
HA-TRIP4
HA-ASCC1
IB: HA

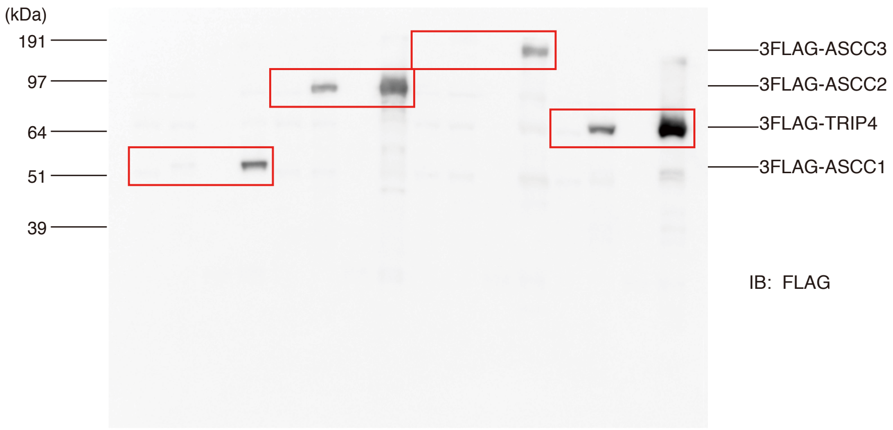
(kDa)
191
97
64
51
39
3FLAG-ASCC3
3FLAG-ASCC2
3FLAG-TRIP4
3FLAG-ASCC1
IB: FLAG

Fig. S4

4A

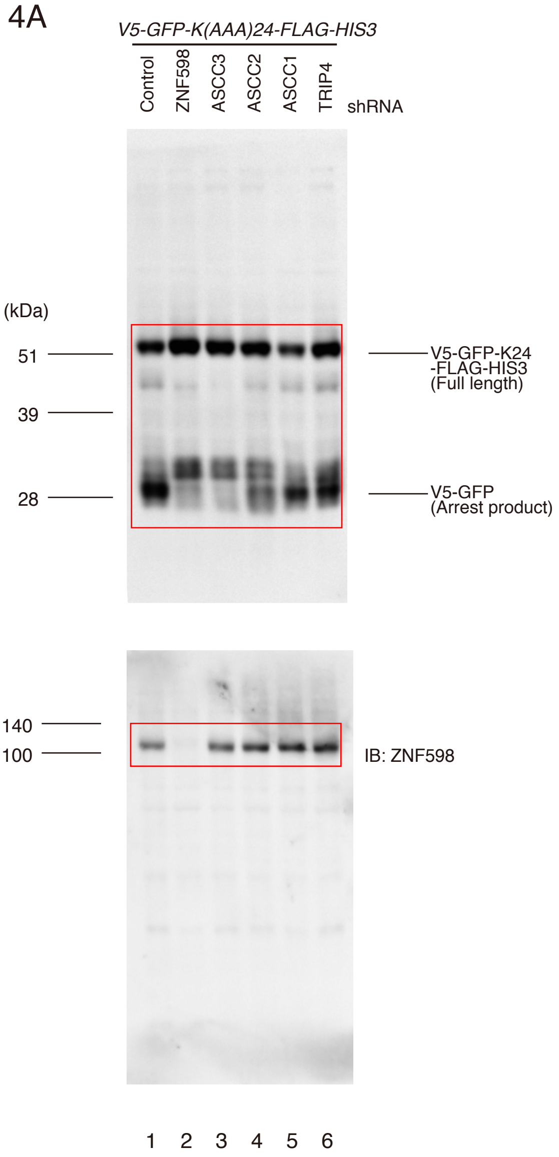

V5-GFP-K(AAA)24-FLAG-HIS3
Control
ZNF598
ASCC3
ASCC2
ASCC1
TRIP4
shRNA
(kDa)
51
39
28
V5-GFP-K24
-FLAG-HIS3
(Full length)
V5-GFP
(Arrest product)
140
100
IB: ZNF598
1 2 3 4 5 6

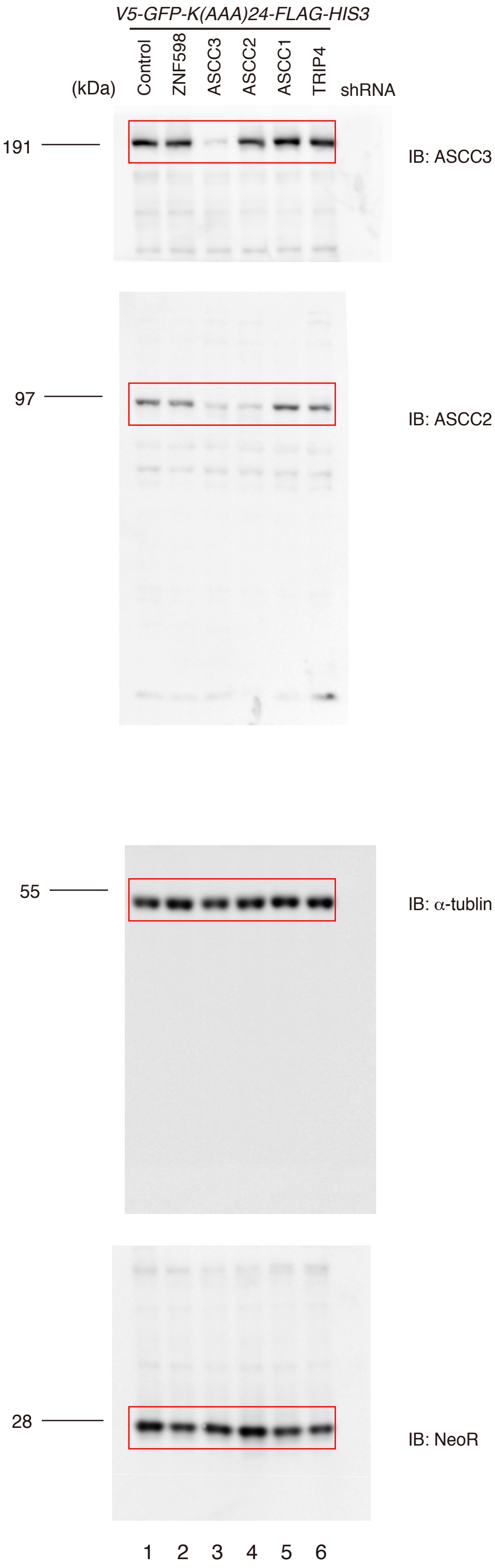

V5-GFP-K(AAA)24-FLAG-HIS3
(kDa)
Control
ZNF598
ASCC3
ASCC2
ASCC1
TRIP4
shRNA
191
IB: ASCC3
97
IB: ASCC2
55
IB: α-tublin
28
IB: NeoR
1 2 3 4 5 6

Fig. S5

4B-D

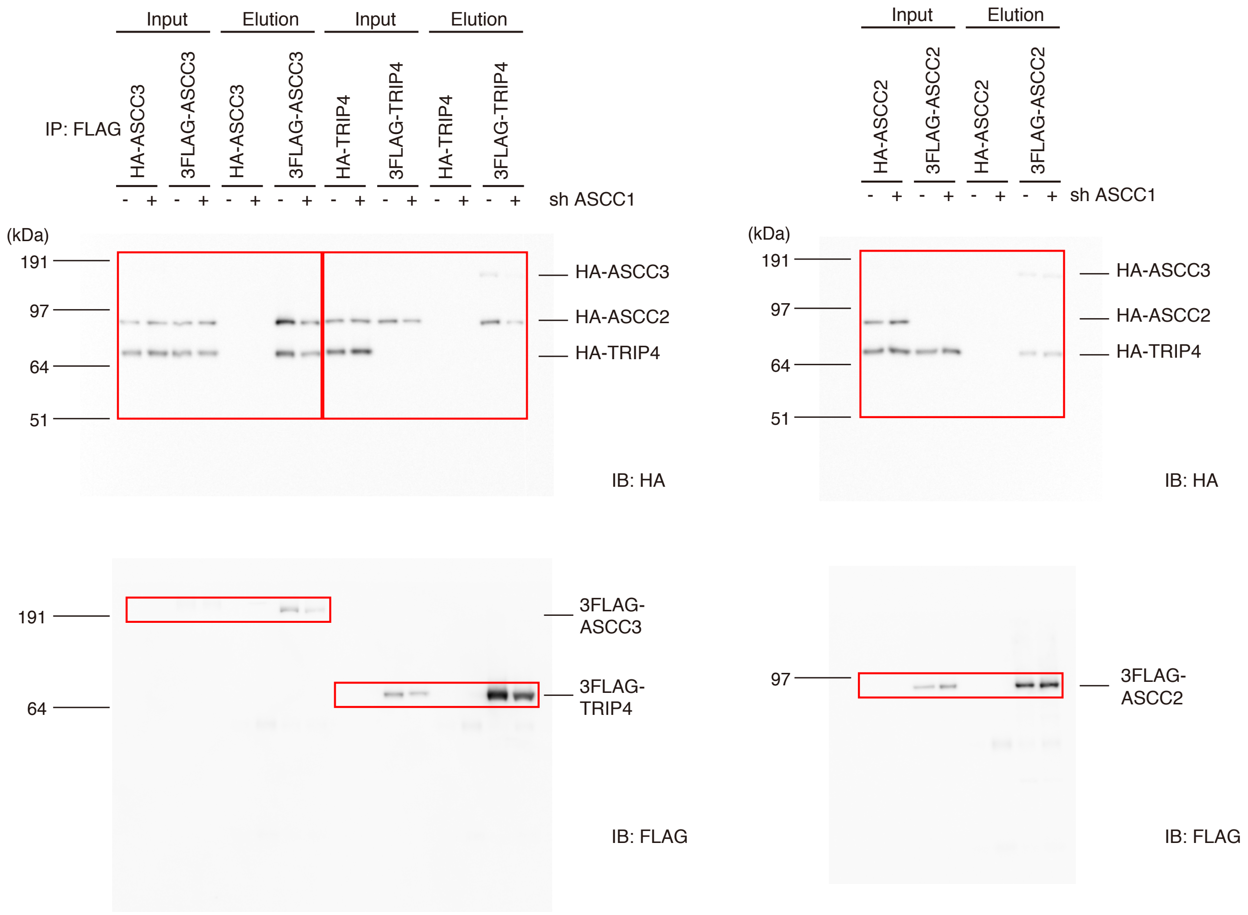

Input
Elution
Input
Elution
IP: FLAG
HA-ASCC3
3FLAG-ASCC3
HA-ASCC3
3FLAG-ASCC3
HA-TRIP4
3FLAG-TRIP4
HA-TRIP4
3FLAG-TRIP4
- + - + - + - + - + - + - + - +
sh ASCC1
(kDa)
191
97
64
51
HA-ASCC3
HA-ASCC2
HA-TRIP4
IB: HA
191
64
3FLAG-ASCC3
3FLAG-TRIP4
IB: FLAG
Input
Elution
HA-ASCC2
3FLAG-ASCC2
HA-ASCC2
3FLAG-ASCC2
- + - + - + - +
sh ASCC1
(kDa)
191
97
64
51
HA-ASCC3
HA-ASCC2
HA-TRIP4
IB: HA
97
3FLAG-ASCC2
IB: FLAG

Fig. S6

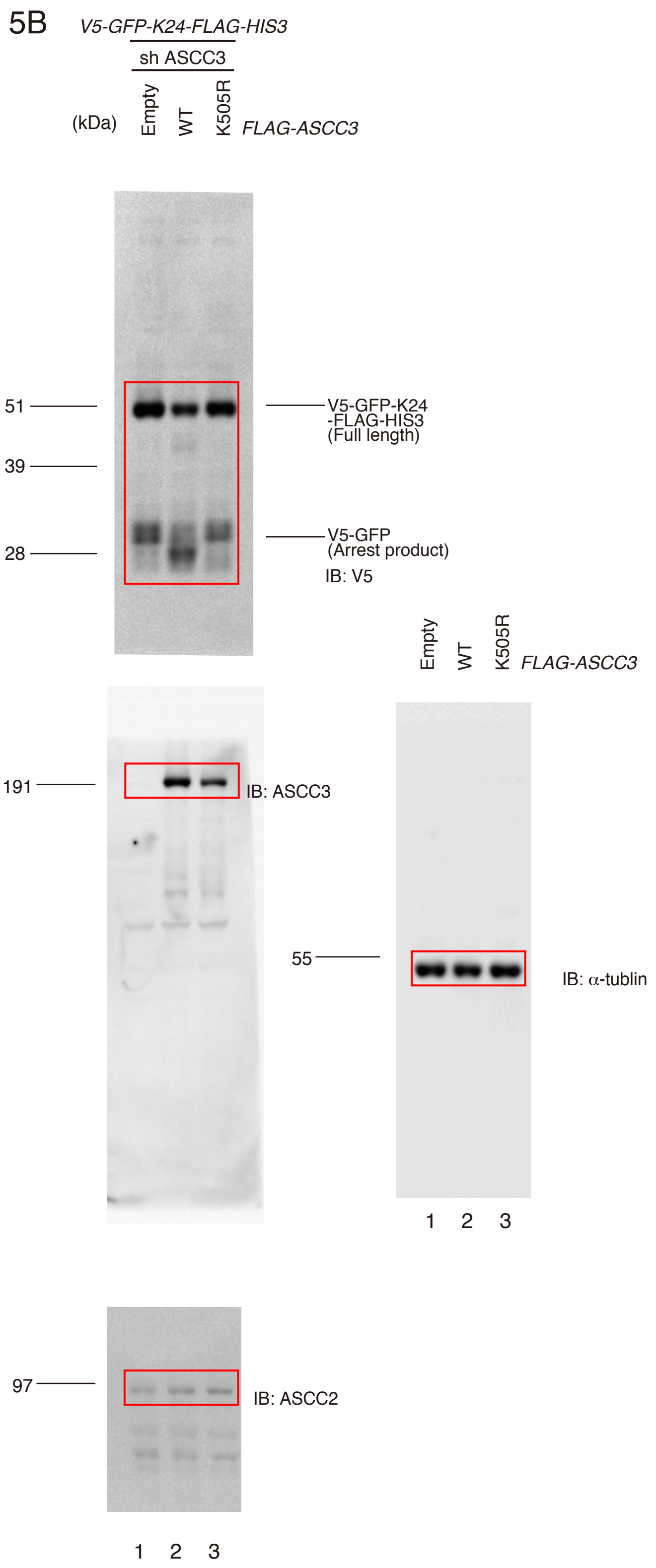
5B
V5-GFP-K24-FLAG-HIS3
sh ASCC3
(kDa)
Empty
WT
K505R
FLAG-ASCC3
51
39
28
V5-GFP-K24
-FLAG-HIS3
(Full length)
V5-GFP
(Arrest product)
IB: V5
191
IB: ASCC3
Empty
WT
K505R
FLAG-ASCC3
55
IB: α-tublin
1 2 3
97
IB: ASCC2
1 2 3

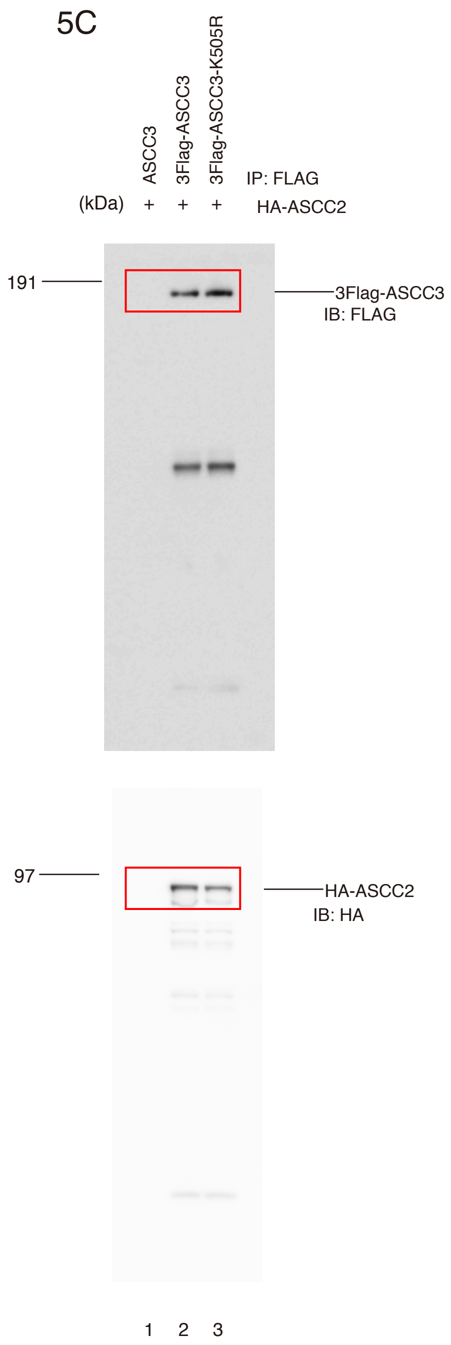
5C
ASCC3
3Flag-ASCC3
3Flag-ASCC3-K505R
IP: FLAG
(kDa)
+ + +
HA-ASCC2
191
3Flag-ASCC3
IB: FLAG
97
HA-ASCC2
IB: HA
1 2 3

Fig. S7

5D

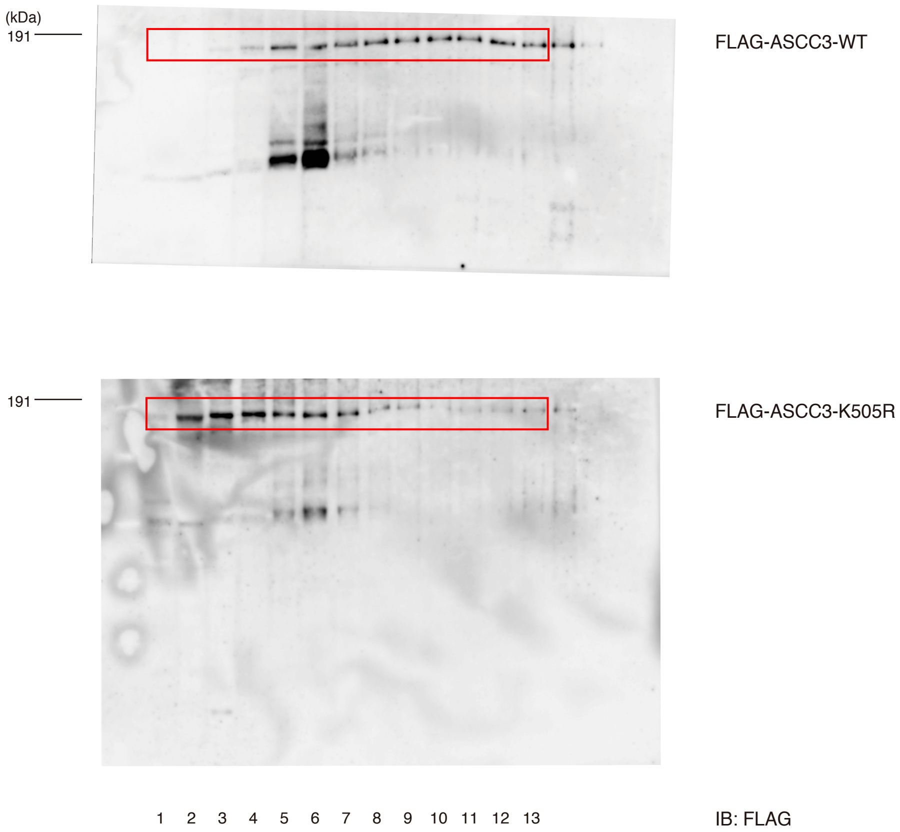
(kDa)
191
FLAG-ASCC3-WT
191
FLAG-ASCC3-K505R
1 2 3 4 5 6 7 8 9 10 11 12 13
IB: FLAG

Fig. S8

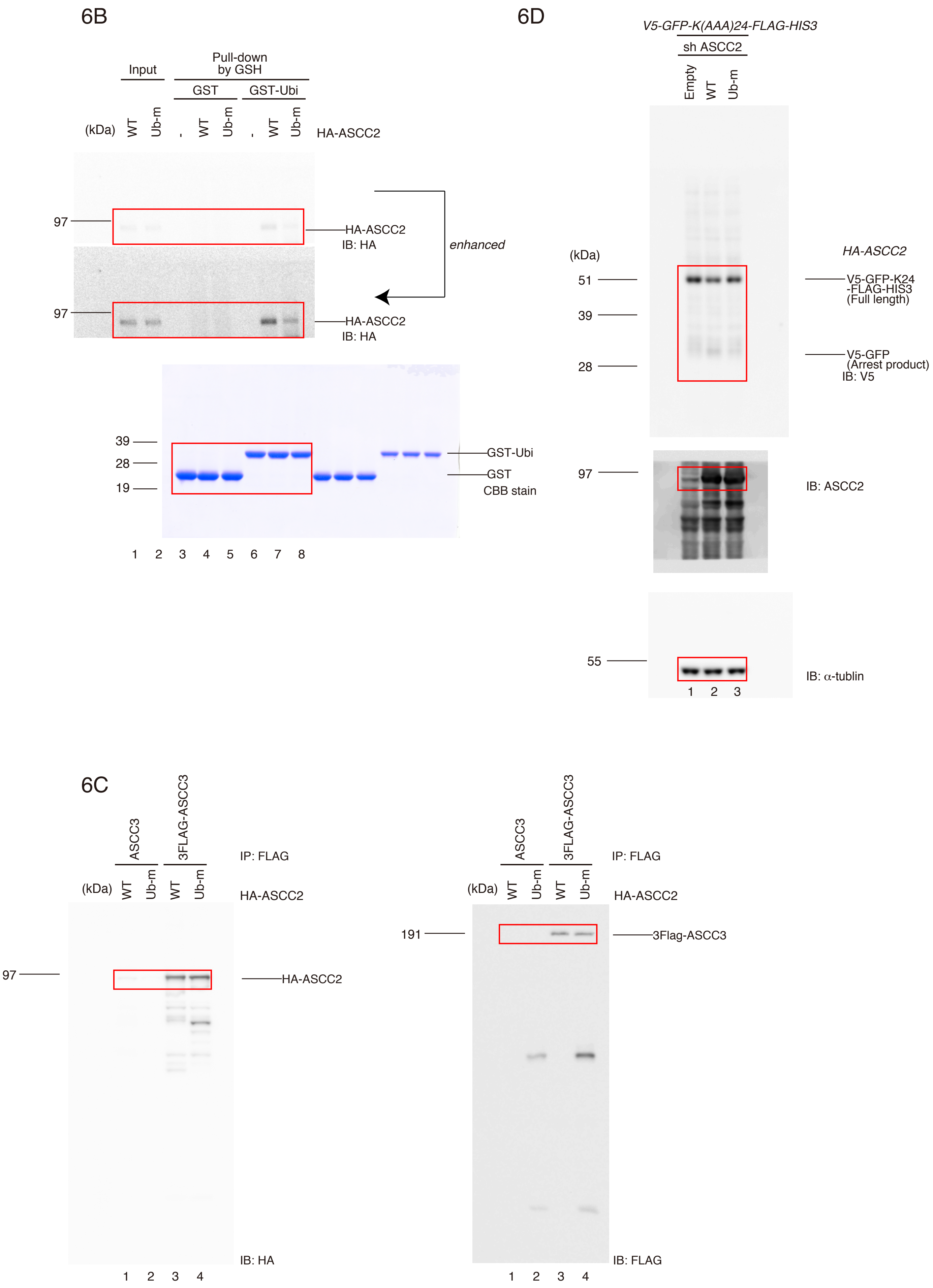

6B
Input
Pull-down
by GSH
GST
GST-Ubi
(kDa)
WT
Ub-m
-
WT
Ub-m
-
WT
Ub-m
HA-ASCC2
97
HA-ASCC2
IB: HA
enhanced
97
HA-ASCC2
IB: HA
39
28
19
GST-Ubi
GST
CBB stain
1 2 3 4 5 6 7 8
6D
V5-GFP-K(AAA)24-FLAG-HIS3
sh ASCC2
Empty
WT
Ub-m
HA-ASCC2
(kDa)
51
39
28
V5-GFP-K24
-FLAG-HIS3
(Full length)
V5-GFP
(Arrest product)
IB: V5
97
IB: ASCC2
55
IB: α-tublin
1 2 3
6C
ASCC3
3FLAG-ASCC3
IP: FLAG
(kDa)
WT
Ub-m
WT
Ub-m
HA-ASCC2
97
HA-ASCC2
IB: HA
1 2 3 4
ASCC3
3FLAG-ASCC3
IP: FLAG
(kDa)
WT
Ub-m
WT
Ub-m
HA-ASCC2
191
3Flag-ASCC3
IB: FLAG
1 2 3 4

Fig. S9

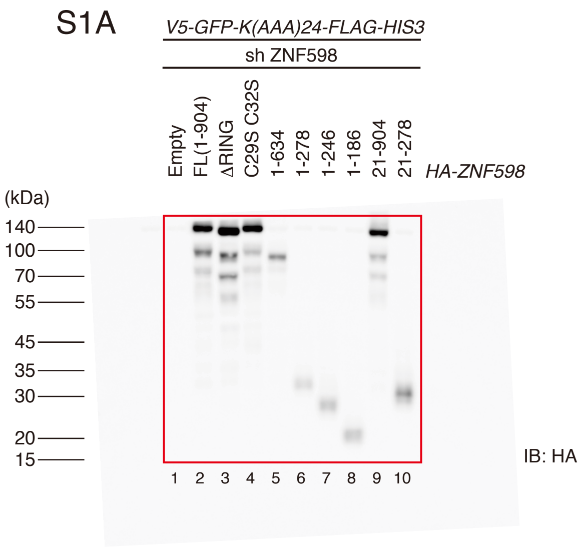
S1A
V5-GFP-K(AAA)24-FLAG-HIS3
sh ZNF598
Empty
FL(1-904)
ΔRING
C29S C32S
1-634
1-278
1-246
1-186
21-904
21-278
HA-ZNF598
(kDa)
140
100
70
55
45
35
30
20
15
1 2 3 4 5 6 7 8 9 10
IB: HA

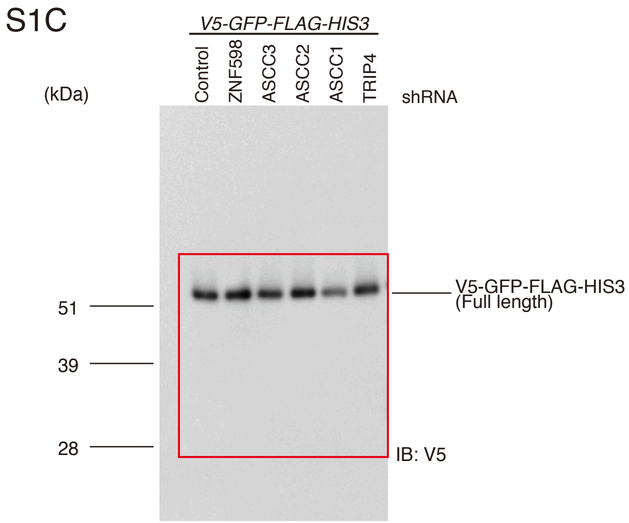
S1C
V5-GFP-FLAG-HIS3
Control
ZNF598
ASCC3
ASCC2
ASCC1
TRIP4
shRNA
(kDa)
51
39
28
V5-GFP-FLAG-HIS3
(Full length)
IB: V5
